# Fake News Detection in Aging During the Era of Infodemic

**DOI:** 10.1093/geroni/igab046.3489

**Published:** 2021-12-17

**Authors:** Didem Pehlivanoglu, Tian Lin, Kevin Chi, Eliany Perez, Rebecca Polk, Barian Cahill, Nichole Lighthall, Natalie Ebner

**Affiliations:** 1 University of Florida, Gainesville, Florida, United States; 2 University of Central Florida, Orlando, Florida, United States

## Abstract

Increasing misinformation spread, including news about COVID-19, poses a threat to older adults but there is little empirical research on this population within the fake news literature. Embedded in the Changes in Integration for Social Decisions in Aging (CISDA) model, this study examined the role of (i) analytical reasoning; (ii) affect; and (iii) news consumption frequency, and their interplay with (iv) news content, in determining fake news detection in aging during the COVID-19 pandemic. Young (age range 18-35 years, M = 20.24, SD = 1.88) and older (age range 61-87 years, M = 70.51, SD = 5.88) adults were randomly assigned to view COVID or non-COVID news articles, followed by measures of analytical reasoning, affect, and news consumption frequency. Comparable across young and older adults, fake news detection accuracy was higher for news unrelated to COVID, and non-COVID fake news detection was predicted by individual differences in analytic reasoning. Examination of chronological age effects further revealed that detection of fake news among older adults aged over 70 years depended on interactions between individual CISDA components and news content. Collectively, these findings suggest that age-related susceptibility to fake news may only be apparent in later stages of older adulthood, but vulnerabilities are context dependent. Our findings advance understanding of psychological mechanisms in fake news evaluation and empirically support CISDA in its application to fake news detection in aging.

